# CT-Derived Features as Predictors of Clot Burden and Resolution

**DOI:** 10.3390/bioengineering11111062

**Published:** 2024-10-24

**Authors:** Quentin Auster, Omar Almetwali, Tong Yu, Alyssa Kelder, Seyed Mehdi Nouraie, Tamerlan Mustafaev, Belinda Rivera-Lebron, Michael G. Risbano, Jiantao Pu

**Affiliations:** 1 Department of Radiology, University of Pittsburgh, Pittsburgh, PA 15260, USA; qauster1@gmail.com (Q.A.); t.mustafaev1994@gmail.com (T.M.); 2 School of Medicine, Marshall University, Huntington, WV 25755, USA; omar.almitwalli@gmail.com; 3 Department of Bioengineering, University of Pittsburgh, Pittsburgh, PA 15213, USA; toy25@pitt.edu; 4 Department of Internal Medicine, School of Medicine and UPMC, University of Pittsburgh, Pittsburgh, PA 15213, USA; keldera@upmc.edu (A.K.); riveralebronbn@upmc.edu (B.R.-L.); risbanomg@upmc.edu (M.G.R.); 5 Division of Pulmonary, Allergy, Critical Care and Sleep Medicine, Department of Medicine, School of Medicine and UPMC, University of Pittsburgh, Pittsburgh, PA 15213, USA; nouraies@upmc.edu; 6 Department of Ophthalmology, University of Pittsburgh, Pittsburgh, PA 15213, USA

**Keywords:** acute and chronic pulmonary embolism (PE), clot burden, PE resolution, AI algorithms

## Abstract

**Objectives**: To evaluate the prognostic utility of CT-imaging-derived biomarkers in distinguishing acute pulmonary embolism (PE) resolution and its progression to chronic PE, as well as their association with clot burden. **Materials and Methods**: We utilized a cohort of 45 patients (19 male (42.2%)) and 96 corresponding CT scans with exertional dyspnea following an acute PE. These patients were referred for invasive cardiopulmonary exercise testing (CPET) at the University of Pittsburgh Medical Center from 2018 to 2022, for whom we have ground truth classification of chronic PE, as well as CT-derived features related to body composition, cardiopulmonary vasculature, and PE clot burden using artificial intelligence (AI) algorithms. We applied Lasso regularization to select parameters, followed by (1) Ordinary Least Squares (OLS) regressions to analyze the relationship between clot burden and the selected parameters and (2) logistic regressions to differentiate between chronic and resolved patients. **Results**: Several body composition and cardiopulmonary factors showed statistically significant association with clot burden. A multivariate model based on cardiopulmonary features demonstrated superior performance in predicting PE resolution (AUC: 0.83, 95% CI: 0.71–0.95), indicating significant associations between airway ratio (negative correlation), aorta diameter, and heart volume (positive correlation) with PE resolution. Other multivariate models integrating demographic features showed comparable performance, while models solely based on body composition and baseline clot burden demonstrated inferior performance. **Conclusions**: Our analysis suggests that cardiopulmonary and demographic features hold prognostic value for predicting PE resolution, whereas body composition and baseline clot burden do not. **Clinical Relevance**: Our identified prognostic factors may facilitate the follow-up procedures for patients diagnosed with acute PE.

## 1. Introduction

Pulmonary embolism (PE) is one of the leading cardiovascular causes of mortality worldwide, surpassed only by stroke and myocardial infarction [1]. Approximately 20% to 25% of PE cases are fatal [2]. Even with treatment, the mortality rate ranges from 8% to 15% [3], leading to over 100,000 deaths annually in the United States [4].

PE can present as acute, chronic, or a combination of both. Acute PE occurs when a clot, typically originating from Deep Vein Thrombosis (DVT) in the lower body [4], dislodges and obstructs a pulmonary artery [5,6]. Chronic PE refers to persistent blockages from unresolved acute PEs [7]. Many patients with acute PE experience ongoing cardiopulmonary symptoms [8]. Nevertheless, the exact risk of progression to chronic PE remains uncertain [9]. Estimates of the resolution rate vary widely, from 50% in one meta-analysis [10] to between 70% and 90% in others [11,12,13]. Typical treatment with anticoagulation medication [8,14] reduces the risk of PE but introduces an inherent risk of bleeding. It is imperative to identify prognostic factors that influence PE resolution and predict susceptibility to disease progression to chronic PE despite treatment [13,15].

Previous studies have investigated the rate of clot resolution by analyzing patient-specific characteristics and relevant CT imaging parameters. These studies found slower resolution in patients with centrally located clots, consolidation, and a history of venous thromboembolism (VTE) [13]. However, they did not consider certain body composition factors. Another study indicated that the absence of previous Chronic Obstructive Pulmonary Disease (COPD), provoked PE, reduced vessel obstruction, and early treatment were associated with a lower risk of residual thrombosis [16]. However, this study did not account for body composition or other cardiopulmonary biomarkers and concluded that the prognostic factors associated with PE resolution on follow-up CT Angiography (CTA) were unclear. Few investigations have been conducted on the combined influence of clot volume, distribution, body composition, and cardiopulmonary vasculature on either clot burden or PE resolution. The feasibility of identifying patients prone to developing chronic PE based on such biomarkers remains unknown.

We propose leveraging artificial intelligence (AI) algorithms to quantitatively and comprehensively analyze a range of image biomarkers and explore the potential associations between body composition, cardiopulmonary vasculature, and two primary outcomes: (1) clot burden and distribution and (2) PE resolution. Artificial intelligence, in particular deep learning methods, have been applied with great success in thoracic radiology, in particular to the detection of lung and heart diseases [17]. The use of convolutional and attention-based models is well suited to image-based thoracic data. These applications include the detection and evaluation of clot burden in patients with pulmonary embolism [18,19,20]. Our goal is to identify novel diagnostic and prognostic CT-derived imaging biomarkers linked to PE resolution and the development of chronic PE.

## 2. Materials and Methods

### 2.1. Study Cohort

We assembled a retrospective cohort incorporating 96 chest CT pulmonary angiogram (CTPA) scans acquired from 45 patients diagnosed with acute PE. These patients were referred to the University of Pittsburgh Medical Center (UPMC) between 2018 and 2022 due to exertional dyspnea. Following diagnostic CTPA scans confirming acute PE, all patients received standard treatment. Among them, 29 patients were classified as having chronic PE based on persistent perfusion defects on a ventilation–perfusion (VQ) scan and/or chronic occlusive clot on subsequent CTPA scans. The remaining 16 patients without these findings were classified as resolved cases.

Three patients who did not undergo a VQ scan were included based on CTPA findings: one showed complete resolution (resolved), one displayed a linear filling defect in pulmonary artery branches (chronic), and one exhibited extensive and worsening emboli (chronic). Additionally, one patient had their initial CT scans performed after their initial VQ scan; however, both a follow-up VQ scan and follow-up CT scans indicated chronic PE. The median and mean durations between the date of the initial CT scan and the VQ were 169 and 364 days, respectively. For follow-up CT scans, the median and mean durations from the initial CT scan were 110 and 358 days, respectively.

### 2.2. CT Image Features Obtained with AI Algorithms

CTPA scans were performed using various GE Medical Systems scanners, including the LightSpeed Pro 16, Optima CT660, LightSpeed VCT, and Discovery CT750 HD models (Milwaukee, Wisconsin, USA). Patients were positioned feet-first and supine (FFS) during the scans. The CT images were reconstructed with a 512 × 512 matrix, and slice thickness varied between 0.625 mm and 1.25 mm. Key scanning parameters included tube voltages ranging from 100 to 120 kV and tube currents averaging between 342 and 598 mAs, ensuring high-quality imaging across different patient conditions and scanner setups.

Our AI algorithms were developed and validated using images from diverse sources [21,22,23,24,25,26,27]. The PE detection and segmentation algorithm [21], in particular, was trained on the RSNA-PE dataset (*n* > 7000) and incorporates a differential geometric approach. The geometric characteristic ensures the algorithm’s robustness, minimizing sensitivity to image noise and artifacts caused by varying imaging protocols. As a result, the system maintains high performance across different datasets and clinical environments, ensuring reliable detection under diverse imaging conditions.

**Figure 1:**
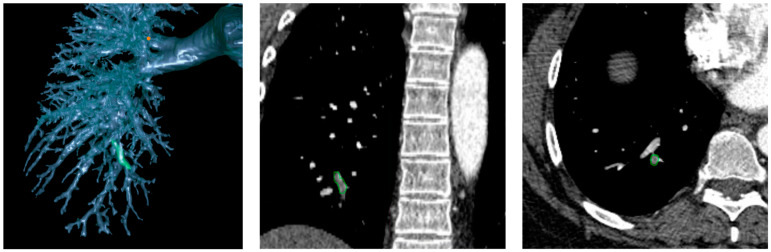
Case from a patient with PE. Green indicates clot locations.

We computed three groups of image features from the CTPA scans in our cohort:

(1) PE characteristics. Our novel AI algorithm automatically identified and segmented isolated PE regions depicted on the CTPA scans [21]. This algorithm was trained on the RSNA Pulmonary Embolism CT Dataset (RSNA-PE) (7279 scans) [21] and validated with 91 independently manually annotated CTPA scans. Based on the segmentation of PE regions, we analyzed clot volumes and their distribution across lung segments and lobes [22,28]. The PE volumes were consolidated at the lobes and the entire lung levels to serve as an index of total clot burden. Figure 1 shows an example of clot locations;
(2) Body composition tissues. We developed a 3-D convolutional neural network (CNN) [25] to automatically segment five body tissues depicted on CT images: visceral adipose tissue (VAT), subcutaneous adipose tissue (SAT), intermuscular adipose tissue (IMAT), skeletal muscle (SM), and bones. Unlike most existing algorithms focusing on abdominal CT scans that typically segment 1–3 types of body tissues based on the cross-sectional area of only a single or a few image slices [29,30,31,32,33], such as at the third cervical (C3) vertebra or the third or fourth lumbar (L3 or L4) vertebra, our AI algorithm volumetrically segments these tissues across various body regions. This algorithm was used to segment and quantify volume, mass, and density of these five tissues from CTPA scans [34];
(3) Cardiopulmonary characteristics. We developed algorithms to outline the pulmonary vascular tree and cardiac silhouette depicted on CT scans [26,35]. Using lung volume segmentation [27], we subclassified the pulmonary vasculature into extra- and intrapulmonary arteries and veins. This allowed us to quantify and compare the individual volumes and densities of these anatomical segments, providing a descriptive ratio between arterial and venous volumes. We calculated vascular volumes at various scales based on cross-sectional area, specifically <5 mm^2^ (BV5) and between 5 and 10 mm^2^ (BV10). By leveraging automated algorithms for lung and airway segmentation [27,36], we computed lung and airway volumes and derived the airway-to-lung volume ratio. Additionally, we quantified emphysematous changes using the density mask method [37] and measured portal vein (PV) diameter, aorta (A) diameter, and their ratio (PV/A).

### 2.3. Statistical Analyses

Statistical analyses were performed to investigate the relationship between patient demographics, CT-derived biomarkers, and two outcomes: PE clot burden (measured by volume) in the “clot burden analysis” (*n* = 45, OLS regression) and PE resolution in the “resolution analysis” (*n* = 96, Logistic regression). Missing values were removed. Numerical features were standardized.

Feature selection was conducted using Lasso regularization within a repeated cross-validation (CV) framework with 5 folds and 10 repetitions, each employing different randomizations. The regularization hyperparameters were adjusted iteratively for each analysis so that each regression model included between one and (n/10) independent variables to ensure at least 10 observations per independent variable. Specifically, if the number of selected features either remained at zero or exceeded the cap, the range of the regularization parameter array was incrementally adjusted upwards and downwards to search for the optimal hyperparameter. After regularization, features with non-zero coefficients were included in the multivariate models—a form of a “relaxed lasso” procedure [38,39] with the hyperparameter γ = 0.

Clot burden analysis was evaluated using mean R2 and RMSE, while resolution analysis was assessed using the mean area under the receiver-operator curve (ROC-AUC) and Brier Score, both calculated from a 5-fold CV. The ROC curves were compared using DeLong’s test [40]. The regression coefficients statistically significant at a 95% confidence level were emphasized.

Data preprocessing, feature selection, regression analyses, and model evaluation were performed in Python 3.10 and R 4.3.1.

## 3. Results

### 3.1. CT-Derived Features

The summary statistics of demographics and CT-derived features in our cohort (*n* = 45) are shown in Table 1. The cohort is predominantly female (57.8%) and white (88.9%). The cohort’s characteristics are largely balanced across the resolved and chronic groups, with the exceptions that the chronic group was younger and more heavily female.

### 3.2. Clot Burden Analysis

Table 2 displays the univariate analysis results on the association between CT-derived features and clot burden across multiple lobes. Body tissue compositions, including bone, muscle, and visceral fat (both mass and volume), as well as cardiopulmonary characteristics including aorta diameter, extra- and intrapulmonary vein volumes, heart volume, and PV/A, exhibited statistically significant positive relationships with clot burden. Conversely, cardiopulmonary characteristics including artery–vein ratio, BV10 and BV5, extrapulmonary artery volume, PV diameter, and PV/A demonstrated a negative relationship with clot burden. Male gender was positively associated with clot burden across multiple lobes. Table S1 includes variables showing statistically significant differences by gender.

To investigate the impact of gender on clot burden across multiple lobes, additional univariate analyses were conducted with gender controlled for (Table S2). Body tissue compositions, including BMI, bone mass, muscle mass and volume, and VFAT mass and volume, showed statistically significant positive relationships with clot burden across multiple lobes. Bone density exhibited a negative relationship with clot burden in the right inferior lobe, while IFAT density showed a negative relationship with clot burden in the central artery. Cardiopulmonary characteristics, including aorta diameter, heart volume, intrapulmonary vein volume, and PV/A, displayed positive associations with clot burden across multiple lobes. Artery–vein ratio, BV10 and BV5, extrapulmonary artery volume, PV diameter, and PV/A were negatively associated with clot burden across multiple lobes.

The multivariate analysis (Table 3) highlighted that bone mass displayed a positive association with total clot burden and central artery clot, without demonstrating a specific predisposition towards individual lobes. Artery–vein ratio, BV5, extrapulmonary arterial volume, and PV/A exhibited negative relationships with clot burden across multiple lobes. Heart volume was positively associated with total clot burden across multiple lobes. Mean CV R2 and RMSE values are provided for each model.

### 3.3. Resolution Analysis

Mann–Whitney U statistical tests were applied to assess distribution differences between resolved and chronic groups. The chronic group exhibited a younger average age and a higher proportion of female patients. Both groups were predominantly composed of white individuals, with the resolved group exclusively consisting of white patients. No statistically significant differences were found between the groups in height, weight, or BMI. The resolved group exhibited a lower airway ratio, higher airway volume, and larger aorta diameter on average (Table 1).

**Figure 2:**
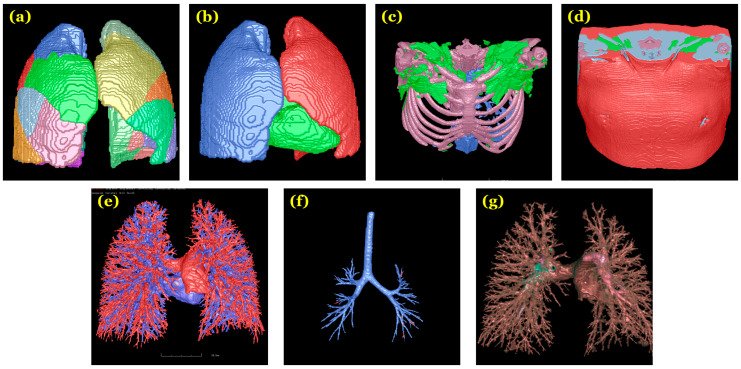
Illustrative images from which features were derived: (**a**) pulmonary segments; (**b**) heart and lungs; (**c**) bone, intermuscular fat (green), and visceral fat (blue); (**d**) subcutaneous fat (red) and muscle (blue); (**e**) arteries (red) and veins (blue); (**f**) central artery; and (**g**) clot regions.

The progression of clot burden relative to the date of the VQ scan was analyzed for each patient. Both groups presented large clots on the initial scans, followed by significant reductions in clot size over time (Figure 2). Despite substantial clot reduction, chronic patients (e.g., PE 6, 24, and 51) still exhibited residual webs and evidence of chronic PE on VQ scans [7,41]. No distinctive patterns were identified to distinguish resolved from chronic PE. Therefore, univariate and multivariate analyses using baseline clot measurement were performed.

The univariate analysis (Table 4) showed no significant relationship between body tissues and PE resolution, regardless of age or gender controls. Without any controls, aorta diameter, heart volume, age, and male gender were positively associated with PE resolution, while airway ratio was negatively associated. When controlling for age, airway ratio (negative correlation) and heart volume (positive correlation) were significantly associated with PE resolution. When controlling for gender, airway ratio (negative correlation) and aorta diameter (positive correlation) were significantly associated with PE resolution.

**Figure 3:**
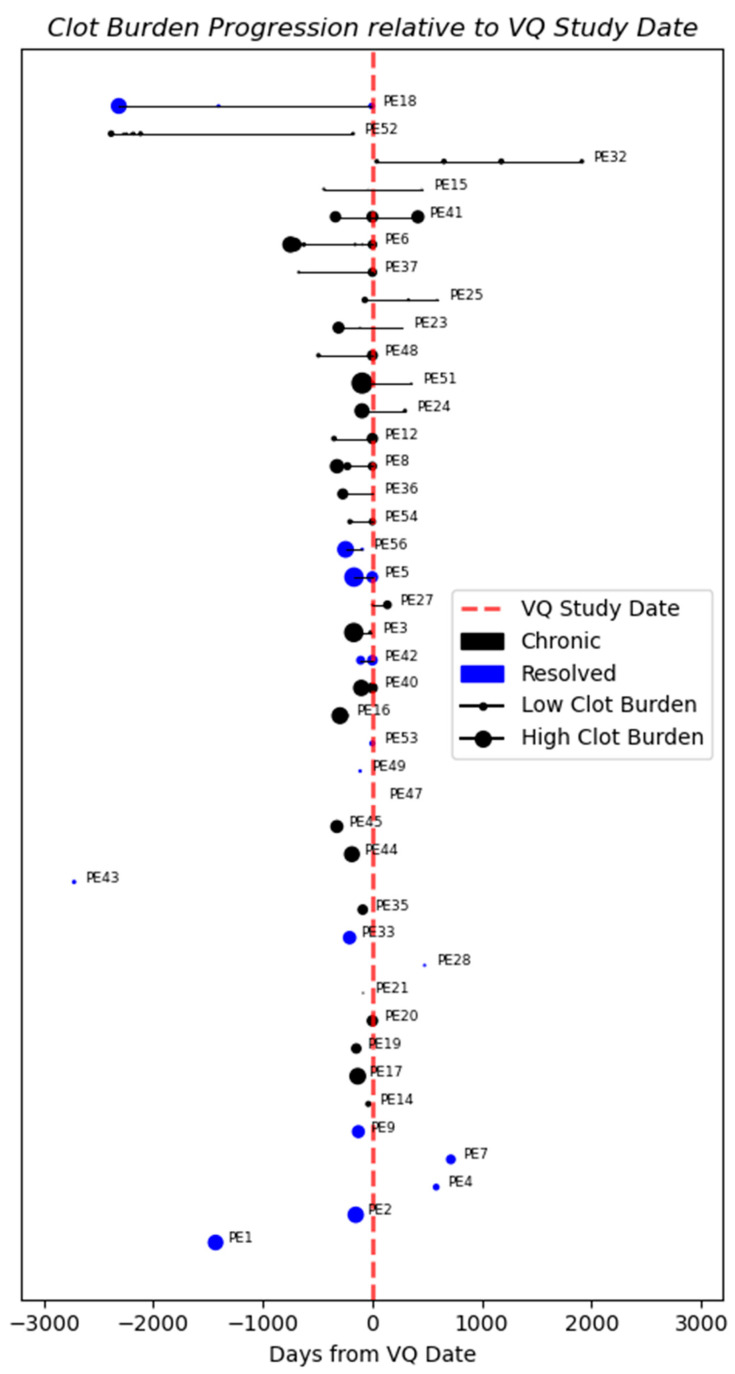
Clot burden progression relative to VQ study date. Patients without a VQ scan were excluded. Larger markers indicate scans with higher measured clot volumes. Markers to the left of the dotted red line represent scans taken prior to VQ study date.

In the multivariate models (Figure 3; Table 4), the demographic model (denoted as “Demo”) achieved a CV AUC of 0.73 (95% CI: 0.57–0.89), with both age and gender positively associated with PE resolution. The body composition model (denoted as “Body”) achieved a CV AUC of 0.65 (95% CI: 0.49–0.82) and included bone density and VFAT density features. However, neither of these features reached statistical significance. The cardiopulmonary model (denoted as “Cardio”) attained a CV AUC of 0.83 (95% CI: 0.71–0.95), with airway ratio negatively associated with PE resolution, and aorta diameter and heart volume positively associated with PE resolution. The model of clot volumes (denoted as “Clot”) achieved a CV AUC of 0.48 (95% CI: 0.30–0.66) and included clot volumes from the left superior, right inferior, and left inferior lobes, yet none of these variables displayed a statistically significant relationship with PE resolution.

**Figure 4:**
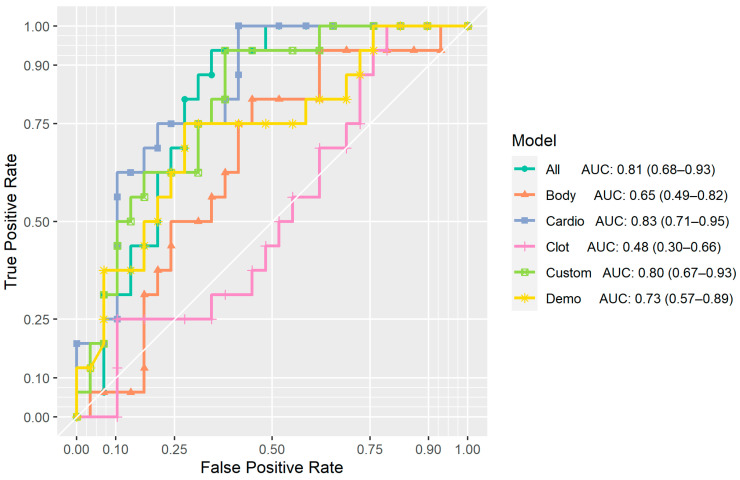
ROC-AUC curves for multivariate models. Curves, scores, and confidence intervals were computed from predicted probabilities from 5-fold cross-validation.

As shown in Figure 4, the model of all variables (denoted as “All”) reached a CV AUC of 0.81 (95% CI: 0.68–0.93) and included airway ratio, aorta diameter, heart volume, and age. Airway ratio was negatively associated with PE resolution, whereas heart volume exhibited a positive association. A custom composite model (denoted as “Custom”) model achieved a CV AUC of 0.80 (95% CI: 0.67–0.93), with airway ratio negatively associated with PE resolution, and aortic diameter and heart volume positively associated with PE resolution.

DeLong’s test for model-to-model comparisons showed statistically significant differences (95% confidence level) in ROC curves between the following pairs: Cardio vs. Body (*p*-value: 0.03), Cardio vs. Clot (*p*-value: <0.01), All vs. Clot (*p*-value: <0.01), and Custom vs. Clot (*p*-value: 0.01) (Table 5).

## 4. Discussion

We conducted a comprehensive investigation of the associations between CT-derived features, clot burden, and PE resolution. To our knowledge, our study is the first to explore the potential relationship between various body tissue compositions, cardiopulmonary vasculature, and clot burden across different pulmonary lobes using radiographic data. We used gold-standard diagnostic confirmation from VQ scans and subclassification based on CTPA as ground-truth labels to distinguish between resolved and chronic PE. By combining these labels with our CT-derived features, we analyzed how these features affect PE resolution. Unlike previous studies that only considered CT-derived features like clot volume and distribution [13,16], we went further by accounting for the combined influence of CT-derived body compositions and cardiopulmonary features, such as quantitative airway and vessel measures.

Our analysis demonstrated the prognostic utility of cardiopulmonary and demographic features as potentially informative tools for predicting PE resolution. Among the multivariate resolution analyses, the demographics (Demo), cardiopulmonary (Cardio), and composite (Composite) models exhibited the highest prognostic value. In contrast, models derived from baseline clot burden volumes (Clot) and body composition (Body) did not perform well, with the Clot model performing the worst among all three (Table 5, Figure 3).

To test the added value of clot burden in a predictive cardiopulmonary model, we incorporated clot burden in the left superior lobe (the only clot feature remaining after feature selection) into a model (Custom) containing the features airway ratio, aortic diameter, and heart volume. However, this model failed to yield any improvement in AUC compared to the Cardio model. These findings align with previous studies suggesting that, although clot burdens can indicate the severity of an episode and treatment effectiveness, they are not reliable indicators of right ventricular (RV) failure or patient mortality [42].

In our cohort, some patients showed significant reductions in clot burden on CT but were still classified as chronic based on VQ findings and residual webs on CT (Figure 2). The poorer performance of the clot burden model contradicts previous studies that used clot characteristics to predict PE resolution speed [13]. This discrepancy may be because they differentiated clot location as central vs. peripheral, whereas we aggregated clot burden by pulmonary lobe.

Although the Cardio, All, and Custom models outperformed the Demo model, the Demo model’s relatively strong performance suggests that these variables capture similar information useful for classification.

Gender plays a significant role in both clot burden and PE resolution within our cohort, underscoring its discriminative value. Our results showed that men exhibited a higher initial clot burden than women (Table 2 and Table S1), yet a higher likelihood of achieving PE resolution (Table 4), which contradicts our initial expectations. Joint evaluation of clot burden and resolution analyses may shed light on these observations. For instance, factors such as aorta diameter and heart volume were associated with both a higher likelihood of resolution and higher clot burden (Table 2). Additionally, the higher representation of women in chronic PE development may be influenced by provoking factors such as oral contraceptive use, which are known to elevate the risk of VTE, including PE, by affecting coagulation factors and blood clotting mechanisms [43]. We also observed that men in our study exhibited higher average values of aorta diameter and heart volume than women (Table S1) and were more likely to experience resolution. One possible explanation for this observation could be attributed to the effects of aorta diameter and heart volume on blood flow and hemodynamics. Increased blood flow may help dislodge the clot or prevent further clot accumulation and propagation, whereas reduced blood flow and stasis could allow for the accumulation of procoagulant proteases leading to further clotting [44].

The significance of gender aligns with the existing literature. Studies suggest that women with PE are more likely to exhibit RV dysfunction [45], a potential indicator of chronic PE on CTPA [3]. Additionally, studies have reported a higher risk of PE-related mortality in women, particularly in hemodynamically stable patients, despite a lower age- and sex-adjusted incidence of PE in women [46]. It is noteworthy that women receiving treatment with anticoagulants have shown a higher risk of bleeding, requiring careful monitoring and potentially differing treatment approaches for acute PE [47]. However, we lacked treatment information to analyze any differences in administering anticoagulants for this study. Despite appearing counterintuitive (given that age is a risk factor for both genders), the statistically significant positive association between age and PE resolution in our study may be explained by the tendency for women diagnosed with PE to be older than men [46,47,48]. Our findings, especially those regarding the association between cardiopulmonary features and PE resolution, could provide insights into the gender-related disparities observed in prior research.

Our analyses provide both etiologic and prognostic insights, partly due to our feature selection method. To ensure interpretability and statistical significance, we balanced the number of features in our models, aiming for a minimum of 10 outcome events per predictor variable (EPV) to avoid biased estimates [49,50,51]. We used Lasso regularization within a cross-validation framework, limiting the number of features based on the target EPV of 10. After feature selection, we conducted regression analysis on the retained features and reported coefficients and *p*-values. This method resembles a “relaxed lasso” procedure [39], with the hyperparameter γ = 0. This approach simplifies the model while dealing with a large number of features, thereby improving prediction accuracy [38]. The value of γ determines the weighting between regularized and maximum likelihood estimation coefficients (1 - γ) [39], balancing model complexity and interpretability. Tuning of γ and making statistical inferences based on these coefficients will require further investigation.

There are several limitations of our study that are worth noting. First, our sample size was small (*n* = 45 for resolution analysis and *n* = 96 for clot burden analysis). Furthermore, conducting feature selection, model training, and evaluation on the same dataset can introduce potential biases. To mitigate these concerns, we employed repeated K-fold cross-validation for both feature selection and model evaluation. Nonetheless, the small sample size limited our ability to analyze and control for other unobserved variables. For instance, as we discuss above, sex may be associated with other features of interest which we capture, such as age, body composition, and cardiopulmonary features, as well as features that we did not have access to, such as oral contraceptive use or menopause. Based on the age distribution of the cohort, it is possible that there could have been a blend or pre- and post-menopausal women, which may affect cardiopulmonary disease. However, we did not have access to this variable, which is a limitation of our study. The inclusion of these features in a larger study presents an opportunity for additional research, in which we could analyze male and female subgroups. Second, our study included one instance where the first CT scan was conducted after the VQ scan, and not all patients had VQ scans to determine PE resolution (*n* = 3). Thirdly, our study cohort included patients already diagnosed with acute PE, and, therefore, our findings should not be extrapolated to predict the risk of acute PE using the aforementioned biomarkers. Finally, our study cohort was from a single center and would benefit from external validation using data from other locations. Given these limitations, future work is needed to establish a deeper understanding of the relationship between body composition, cardiopulmonary features, and PE by collecting a large cohort acquired from multiple sites.

## 5. Conclusions

Our study investigated the relationship between body composition, cardiopulmonary features, clot burden, and PE resolution in a patient cohort. We observed that cardiopulmonary variables, particularly those related to arterial and venous diameters, offered predictive power for clot burden and resolution. In contrast, body composition variables and clot burden measures did not demonstrate such predictive power. This highlights the significance of incorporating both demographic and cardiopulmonary variables when assessing PE severity and potential resolution.

## Figures and Tables

**Table 1:** Demographics and CT-derived features for resolved vs. chronic groups (*n* = 45). Standard error (SE) and count (%) for continuous and categorical variables, respectively. *p*-values ≤ 0.05 from Mann–Whitney U tests are highlighted.

Feature	Resolved (*n* = 16)	Chronic (*n* = 29)	All (*n* = 45)	*p*-Value
**Demographic**				
Age	60.4 (3.1)	48.5 (2.9)	52.8 (2.3)	0.02
Race (white)	16 (100.0%)	24 (82.8%)	40 (88.9%)	0.21
Gender (female)	5 (31.3%)	21 (72.4%)	26 (57.8%)	0.02
**Clot Burden**				
Right Superior Lobe	1.19 (0.29)	0.95 (0.19)	1.04 (0.16)	0.69
Left Superior Lobe	0.77 (0.23)	1.14 (0.34)	1.01 (0.24)	0.99
Right Middle Lobe	0.37 (0.16)	0.35 (0.08)	0.36 (0.08)	0.99
Right Inferior Lobe	0.73 (0.21)	0.39 (0.14)	0.51 (0.12)	0.29
Left Inferior Lobe	0.64 (0.17)	0.66 (0.14)	0.65 (0.11)	0.90
Central Artery	6.78 (1.99)	7.32 (1.58)	7.13 (1.23)	0.93
Total Clot Burden	10.47 (2.65)	10.82 (2.03)	10.69 (1.59)	0.93
**Body Composition**				
BMI	32.9 (1.4)	34.6 (1.5)	34.0 (1.1)	0.56
Height (cm)	175.6 (3.0)	171.1 (1.4)	172.7 (1.4)	0.26
Weight (kg)	102.2 (5.8)	101.8 (4.7)	101.9 (3.6)	0.84
Bone Density	307.8 (14.8)	340.4 (11.1)	328.8 (9.1)	0.13
Bone Mass	2.40 (0.13)	2.31 (0.12)	2.35 (0.09)	0.35
Bone Volume	1.63 (0.09)	1.53 (0.08)	1.57 (0.06)	0.21
IFAT Density	−84.8 (2.1)	−82.7 (1.1)	−83.4 (1.0)	0.48
IFAT Mass	0.72 (0.08)	0.68 (0.06)	0.69 (0.05)	0.77
IFAT Volume	0.69 (0.08)	0.65 (0.06)	0.66 (0.05)	0.75
Muscle Density	32.5 (2.3)	32.7 (2.2)	32.6 (1.6)	0.89
Muscle Mass	5.72 (0.37)	5.51 (0.40)	5.58 (0.28)	0.27
Muscle Volume	4.87 (0.31)	4.70 (0.33)	4.76 (0.24)	0.25
SFAT Density	−97.4 (2.3)	−96.8 (2.1)	−97.0 (1.5)	0.93
SFAT Mass	5.71 (0.89)	6.50 (0.89)	6.22 (0.65)	0.71
SFAT Volume	5.58 (0.88)	6.34 (0.87)	6.07 (0.64)	0.70
VFAT Density	−95.2 (1.3)	−91.1 (1.5)	−92.6 (1.1)	0.08
VFAT Mass	1.63 (0.21)	1.39 (0.26)	1.48 (0.18)	0.20
VFAT Volume	1.59 (0.21)	1.35 (0.25)	1.43 (0.18)	0.20
**Cardiopulmonary**				
Airway Ratio	0.02 (0.01)	0.05 (0.01)	0.04 (0.01)	0.02
Airway Volume	0.06 (0.01)	0.05 (0.00)	0.05 (0.00)	0.04
Aorta Diameter	26.2 (1.1)	22.9 (0.6)	24.0 (0.6)	0.01
Artery-Vein Ratio	0.80 (0.04)	0.81 (0.02)	0.81 (0.02)	0.68
BV10	108.9 (16.6)	123.9 (6.8)	118.6 (7.3)	0.44
BV5	44.5 (8.4)	50.8 (4.0)	48.5 (3.9)	0.33
Emphysema Volume (950HU)	0.00 (0.00)	0.00 (0.00)	0.00 (0.00)	0.07
Extrapulmonary Artery Volume	0.12 (0.01)	0.10 (0.00)	0.11 (0.01)	0.14
Extrapulmonary Vein Volume	0.12 (0.01)	0.12 (0.01)	0.12 (0.01)	0.70
Heart Volume	0.66 (0.03)	0.58 (0.02)	0.61 (0.02)	0.05
Intrapulmonary Artery Volume	0.12 (0.01)	0.12 (0.00)	0.12 (0.00)	0.53
Intrapulmonary Vein Volume	0.16 (0.01)	0.15 (0.01)	0.15 (0.01)	0.30
Lung Volume	4.12 (0.37)	3.68 (0.23)	3.84 (0.20)	0.36
PB Larger 10	74.1 (10.6)	75.2 (6.4)	74.8 (5.5)	0.75
PV Diameter	36.4 (2.1)	34.1 (1.1)	34.9 (1.0)	0.23
PV/A	1.43 (0.10)	1.51 (0.06)	1.48 (0.05)	0.51

**Table 2:** Univariate analysis of CT-derived features and clot burden (*n* = 96). Values represent coefficients (positive value indicates a positive relationship to clot burden; negative values indicate a negative relationship to clot burden). Highlighted cells indicate coefficients with *p*-values ≤ 0.05.

Feature	Right Superior Lobe	Left Superior Lobe	Right Middle Lobe	Right Inferior Lobe	Left Inferior Lobe	Central Artery	Total Clot Burden
**Body Composition**							
BMI	0.12	0.15	0.11	0.01	0.12	0.16	0.17
Bone Density	−0.14	0.14	−0.06	−0.24	−0.20	0.10	0.04
Bone Mass	0.38	0.28	0.42	0.36	0.31	0.30	0.38
Bone Volume	0.41	0.26	0.44	0.40	0.34	0.28	0.37
IFAT Density	0.02	−0.15	0.05	0.08	0.17	−0.26	−0.19
IFAT Mass	0.15	0.21	0.13	0.07	0.09	0.16	0.18
IFAT Volume	0.15	0.21	0.13	0.07	0.08	0.17	0.19
Muscle Density	0.08	0.04	0.10	0.07	0.07	0.02	0.05
Muscle Mass	0.33	0.22	0.39	0.25	0.32	0.22	0.29
Muscle Volume	0.33	0.22	0.39	0.25	0.32	0.22	0.29
SFAT Density	−0.04	−0.17	−0.05	0.07	−0.07	−0.15	−0.14
SFAT Mass	0.11	0.18	0.10	0.04	0.18	0.08	0.12
SFAT Volume	0.11	0.18	0.10	0.04	0.18	0.08	0.12
VFAT Density	−0.12	−0.27	−0.13	−0.13	−0.16	−0.22	−0.24
VFAT Mass	0.37	0.33	0.31	0.41	0.39	0.28	0.37
VFAT Volume	0.37	0.34	0.31	0.41	0.39	0.28	0.37
**Cardiopulmonary**							
Airway Ratio	0.00	0.01	−0.03	0.00	−0.10	0.14	0.10
Airway Volume	0.19	0.06	0.16	0.17	0.16	0.21	0.22
Diameter	0.38	0.12	0.30	0.39	0.34	0.19	0.27
Artery-Vein Ratio	−0.34	−0.38	−0.23	−0.21	−0.18	−0.49	−0.49
BV10	−0.25	−0.25	−0.22	−0.24	−0.37	−0.07	−0.17
BV5	−0.27	−0.24	−0.22	−0.24	−0.34	−0.11	−0.20
Emphysema Volume (950HU)	0.19	0.12	0.16	0.26	0.13	0.18	0.21
Extrapulmonary Artery Volume	−0.14	−0.21	−0.11	−0.26	−0.35	−0.10	−0.17
Extrapulmonary Vein Volume	0.20	0.19	0.16	0.04	−0.04	0.41	0.36
Heart Volume	0.39	0.14	0.38	0.33	0.27	0.11	0.21
Intrapulmonary Artery Volume	−0.08	−0.09	−0.04	−0.15	−0.26	0.10	0.02
Intrapulmonary Vein Volume	0.22	0.18	0.19	0.08	−0.02	0.37	0.34
Lung Volume	0.13	−0.02	0.21	0.13	0.13	0.23	0.21
PB Larger 10	0.26	0.06	0.33	0.24	0.25	−0.02	0.07
PV Diameter	−0.18	−0.21	−0.13	−0.26	−0.31	−0.11	−0.18
PV/A	−0.37	−0.25	−0.26	−0.39	−0.42	−0.21	−0.31
**Demographic**							
Age	0.15	0.04	0.07	0.21	0.17	0.11	0.14
Gender (Male)	0.73	0.36	0.67	0.64	0.64	0.43	0.58

**Table 3:** Multivariate analysis of CT-derived features and clot burden (*n* = 96). Values represent coefficients (positive value indicates a positive relationship to clot burden; negative values indicate a negative relationship to clot burden). Highlighted cells indicate coefficients with *p*-values ≤ 0.05. Values of 0.00 are non-zero but are truncated due to rounding.

Feature	Right Superior Lobe	Left Superior Lobe	Right Middle Lobe	Right Inferior Lobe	Left Inferior Lobe	Central Artery	Total Clot Burden
**Body Composition**							
BMI						0.15	
Bone Density		0.19					
Bone Mass		0.09				0.18	0.19
Bone Volume	0.06		0.14	0.05	0.00		
IFAT Density						−0.22	
Muscle Mass					0.06		
VFAT Density		−0.16					
VFAT Volume	0.00	0.17		0.08	0.15		0.09
**Cardiopulmonary**							
Airway Ratio						0.24	
Diameter				0.20			
Artery-Vein Ratio	−0.33	−0.34	−0.25	−0.17	−0.18	−0.24	−0.32
BV10		−0.18	−0.14		−0.11		
BV5	−0.14					−0.17	−0.19
Emphysema Volume (950HU)	0.22		0.19	0.19			
Extrapulmonary Artery Volume				−0.38	−0.33	−0.40	
Extrapulmonary Vein Volume						0.42	0.27
Heart Volume	0.33		0.23	0.39	0.33		
PB Larger 10	0.13		0.23				
PV Diameter		−0.22					
PV/A	−0.34		−0.19	−0.05	−0.19	−0.10	−0.29
**Mean R-squared**	0.28	0.20	0.28	0.25	0.24	0.36	0.32
**Mean RMSE**	0.81	0.81	0.82	0.87	0.84	0.78	0.79

**Table 4:** Univariate and multivariate analyses of CT-derived features and PE resolution (*n* = 45). Values represent coefficients (positive value indicates a positive correlation to PE resolution; negative values indicate a negative relationship to PE resolution). Highlighted cells indicate coefficients with *p*-values ≤ 0.05. Only features with statistically significant coefficients or those included in multivariate models are shown.

	Univariate Models			Multivariate Models					
Feature	No Controls	Age Control	Gender Control	Demo	Body	Cardio	Clot	All	Custom
**Body Composition**									
Bone Density	−0.55	−0.35	−0.44		−0.55				
VFAT Density	−0.57	−0.38	−0.46		−0.57				
**Cardiopulmonary**									
Airway Ratio	−1.09	−1.02	−1.39			−2.23		−2.17	−1.63
Airway Volume	0.63	0.50	0.17			0.79			
Aortic Diameter	1.22	0.90	1.05			1.73		1.07	2.04
Heart Volume	0.70	0.86	0.32			1.18		1.78	1.41
**Clot**									
Left Superior Lobe	−0.22	−0.34	−0.47				−0.26		−0.80
Right Inferior Lobe	0.37	0.28	0.16				0.68		
Left Inferior Lobe	−0.04	0.02	−0.30				−0.39		
**Demographic**									
Age	0.91			0.90				1.08	
Gender (Male)	1.75			1.70					
**Mean AUC**				0.73	0.65	0.83	0.48	0.81	0.80
**Mean Brier Score**				0.20	0.23	0.17	0.25	0.21	0.18

**Table 5:** DeLong test *p*-values from model-to-model comparisons. Each cell represents the *p*-value from a two-sided DeLong test comparing the models in the row and column. (Highlighted cells indicate coefficients with *p*-values ≤ 0.05).

Model	Body	Cardio	Clot	All	Custom
**Demo**	0.40	0.21	0.05	0.32	0.41
**Body**		0.03	0.18	0.08	0.11
**Cardio**			<0.01	0.53	0.38
**Clot**				<0.01	0.01
**All**					0.91

## Data Availability

The data is available upon request.

## Supplementary Materials

The following supporting information can be downloaded at: https://www.mdpi.com/article/10.3390/bioengineering11111062/s1, Table S1. Gender breakdown of body composition, cardiopulmonary, and clot features. Only features with statistically significant differences (95% confidence level) are shown. *p*-values from Mann-Whitney U tests. Table S2. Univariate analysis of CT-derived features and clot burden (*n* = 96) controlled for gender. Values represent coefficients of the variable of interest (positive value indicates a positive relationship to clot burden; negative values indicate a negative relationship to clot burden). Coefficients for gender are not shown. Highlighted cells indicate coefficients with *p*-values ≤ 0.05.
