# Supplementary material for: CT-Derived Features as Predictors of Clot Burden and Resolution
Source: Bioengineering (Basel). 2024 Oct 24;11(11):1062. doi: 10.3390/bioengineering11111062 (PMC11590948; doi:10.3390/bioengineering11111062)
Supplement: Supplementary file 1 [file bioengineering-11-01062-s001.zip › bioengineering-3226164-supplementary.pdf]

Supplementary Table S1. Gender breakdown of body composition, cardiopulmonary, and clot features. Only features with statistically significant differences (95% confidence level) are shown. P-values from Mann-Whitney U tests.

	Female (n=26)	Male (n=19)	All (n=45)	p-value
**Body Composition**				
Bone Mass	2.10 (0.09)	2.68 (0.14)	2.35 (0.09)	<0.01
Bone Volume	1.39 (0.06)	1.81 (0.10)	1.57 (0.06)	<0.01
Muscle Density	30.1 (2.2)	36.1 (2.1)	32.6 (1.6)	0.05
Muscle Mass	4.79 (0.3)	6.67 (0.4)	5.58 (0.3)	<0.01
Muscle Volume	4.09 (0.3)	5.67 (0.3)	4.76 (0.2)	<0.01
VFAT Mass	1.09 (0.17)	2.01 (0.33)	1.48 (0.18)	0.02
VFAT Volume	1.06 (0.17)	1.95 (0.32)	1.43 (0.18)	0.02
**Cardiopulmonary**				
Airway Volume	0.04 (0.0)	0.07 (0.0)	0.05 (0.0)	<0.01
Aortic Diameter	22.9 (0.6)	25.6 (1.0)	24.0 (0.6)	0.02
Extrapulmonary Vein Volume	0.11 (0.0)	0.14 (0.0)	0.12 (0.0)	<0.01
Heart Volume	0.54 (0.0)	0.69 (0.0)	0.61 (0.0)	<0.01
Intrapulmonary Artery Volume	0.11 (0.0)	0.13 (0.0)	0.12 (0.0)	0.03
Intrapulmonary Vein Volume	0.14 (0.0)	0.17 (0.0)	0.15 (0.0)	<0.01
Lung Volume	3.39 (0.2)	4.44 (0.3)	3.84 (0.2)	0.01
**Clot**				
Right Superior Lobe	0.65 (0.15)	1.56 (0.28)	1.04 (0.16)	<0.01
Right Middle Lobe	0.23 (0.08)	0.53 (0.15)	0.36 (0.08)	0.02
Right Inferior Lobe	0.28 (0.11)	0.82 (0.22)	0.51 (0.12)	0.01
Left Inferior Lobe	0.51 (0.14)	0.86 (0.17)	0.65 (0.11)	0.05

Supplementary Table S2. Univariate analysis of CT-derived features and clot burden (n=96) controlled for gender. Values represent coefficients of the variable of interest (positive value indicates a positive relationship to clot burden; negative values indicate a negative relationship to clot burden). Coefficients for gender are not shown. Highlighted cells indicate coefficients with p-values ≤ 0.05.

	Right Superior Lobe	Left Superior Lobe	Right Middle Lobe	Right Inferior Lobe	Left Inferior Lobe	Central Artery	Total Clot Burden
**Body Composition**							
BMI	0.19	0.18	0.18	0.07	0.18	0.20	0.22
Bone Density	-0.07	0.18	0.01	-0.18	-0.14	0.16	0.11
Bone Mass	0.27	0.27	0.35	0.27	0.20	0.27	0.32
Bone Volume	0.31	0.24	0.38	0.33	0.25	0.25	0.32
IFAT Density	0.01	-0.16	0.04	0.07	0.16	-0.27	-0.20
IFAT Mass	0.15	0.21	0.13	0.07	0.08	0.16	0.18
IFAT Volume	0.15	0.21	0.12	0.07	0.08	0.17	0.18
Muscle Density	-0.03	-0.02	0.00	-0.02	-0.03	-0.04	-0.04
Muscle Mass	0.19	0.18	0.31	0.10	0.22	0.15	0.20
Muscle Volume	0.19	0.18	0.31	0.10	0.22	0.15	0.20
SFAT Density	-0.08	-0.19	-0.09	0.04	-0.11	-0.17	-0.17
SFAT Mass	0.19	0.23	0.18	0.12	0.26	0.14	0.19
SFAT Volume	0.19	0.23	0.18	0.11	0.26	0.14	0.19
VFAT Density	-0.02	-0.24	-0.04	-0.04	-0.08	-0.18	-0.18

	Right Superior Lobe	Left Superior Lobe	Right Middle Lobe	Right Inferior Lobe	Left Inferior Lobe	Central Artery	Total Clot Burden
VFAT Mass	0.27	0.32	0.21	0.35	0.31	0.23	0.31
VFAT Volume	0.27	0.32	0.21	0.35	0.31	0.24	0.31
**Cardiopulmonary**							
Airway Ratio	0.00	0.01	-0.03	0.00	-0.10	0.14	0.10
Airway Volume	-0.05	-0.08	-0.07	-0.05	-0.06	0.13	0.07
Aorta Diameter	0.30	0.07	0.20	0.33	0.26	0.13	0.19
Artery-Vein Ratio	-0.27	-0.36	-0.17	-0.15	-0.11	-0.47	-0.45
BV10	-0.25	-0.25	-0.22	-0.24	-0.37	-0.07	-0.17
BV5	-0.26	-0.23	-0.21	-0.23	-0.33	-0.10	-0.19
Emphysema Volume (950HU)	0.15	0.10	0.12	0.22	0.10	0.16	0.18
Extrapulmonary Artery Volume	-0.21	-0.25	-0.17	-0.33	-0.42	-0.14	-0.23
Extrapulmonary Vein Volume	0.11	0.16	0.08	-0.05	-0.13	0.39	0.31
Heart Volume	0.29	0.08	0.30	0.24	0.16	0.02	0.11
Intrapulmonary Artery Volume	-0.17	-0.14	-0.13	-0.24	-0.35	0.06	-0.05
Intrapulmonary Vein Volume	0.11	0.14	0.09	-0.03	-0.14	0.34	0.28
Lung Volume	-0.01	-0.11	0.09	0.01	0.01	0.18	0.12
PB Larger 10	0.17	0.01	0.26	0.16	0.18	-0.09	-0.01
PV Diameter	-0.21	-0.23	-0.16	-0.28	-0.33	-0.13	-0.20
PV/A	-0.32	-0.23	-0.22	-0.35	-0.38	-0.19	-0.27
